# The Ontogeny of Exploratory Behavior in Male and Female Adolescent Rats (*Rattus norvegicus*)

**DOI:** 10.1002/dev.20386

**Published:** 2009-07-06

**Authors:** Debra A Lynn, Gillian R Brown

**Affiliations:** School of Psychology, University of St AndrewsSouth Street, St Andrews, Fife KY16 9JP, UK E-mail: dal5@st-andrews.ac.uk

**Keywords:** adolescence, exploration, novelty, rats, sex differences

## Abstract

During adolescence, rats gain independence from their mothers and disperse from the natal burrow, with males typically dispersing further than females. We predicted that, if dispersal patterns are associated with responsiveness to novelty, exploratory behavior in novel environments would increase across adolescence, and males would explore more than females. Alternatively, females might explore more than males, if females are more motivated than males to learn about the immediate environment or if females have poorer spatial abilities than males. Twenty-five male and 21 female rats were exposed to two novel environments (open field and elevated plus-maze) during early, mid-, or late adolescence. Total locomotion and amount of exploration directed towards aversive areas increased across adolescence, even when body weight was included as a covariate. Female adolescents locomoted more and spent more time exploring aversive areas than males. Developmental changes in neural function potentially underlie age and sex differences in exploratory behavior. © 2009 Wiley Periodicals, Inc. *Dev Psychobiol* 51: 513–520, 2009.

## INTRODUCTION

“Exploration” has been defined as active investigation (e.g., locomotion) that might lead to an animal gaining information about its environment (Birke & Archer, 1983). Exploratory behavior during adolescence potentially allows youngsters to learn about novel aspects of the environment, to disperse to new territories and to gain the necessary skills for independence (Spear, 2000a,b). Understanding the biological basis of exploratory behavior during adolescence could potentially help us to understand the increase in novelty-seeking and risk-taking behavior that accompanies human adolescence, particularly amongst boys (Arnett, 1992; Byrnes, Miller, & Schafer, 1999; Tapert, Aarons, Sedlar, & Brown, 2001).

However, relatively little is known about the ontogeny of exploratory behavior in a common laboratory species, the rat. The aim of this study was to investigate the ontogeny of exploratory behavior in novel environments in rats across adolescence.

Adolescence in rats encompasses the period from weaning (postnatal day, *pnd*, 21) to early adulthood (pnd 60), and this period can be further divided into early (pnd 21–33), mid- (pnd 34–46), and late adolescence (pnd 47–59) (based on Tirelli, Laviola, & Adriani, 2003). During adolescence, young rats begin to emerge from the natal burrow system, follow their mothers on foraging trips, sleep in nest chambers away from the mother, and eventually disperse from the natal area (Calhoun, 1963). As in mostrodent species (Krebs, Lambin, & Wolff, 2007), dispersal is male-biased in rats, with females typically staying closer to the natal burrow system than males (Calhoun, 1963). In birds, dispersal behavior has been reported to correlate with exploratory behavior in novel environments, such that individuals exhibiting high levels of exploratory behavior will disperse sooner or further than other individuals (Dingemanse, Both, van Noordwijk, Rutten, & Drent, 2003). If dispersal patterns are associated with responsiveness to novel environments more generally, we would predict that exploration in rats would increase across adolescence and be higher in male than female adolescents.

However, several alternative hypotheses can be proposed for how sex differences in dispersal will impact upon exploratory behavior in the laboratory. Female rats might exhibit a higher motivation to learn about the immediate environment than males, if females are more likely than males to remain in the local area surrounding the natal burrow system. Learning features of the local environment might not benefit young males that will disperse to new territories. Studies of sex differences in spatial learning ability in rats have shown that females are more likely than males to rely on landmark cues in the environment to solve spatial tasks (e.g., Tropp & Markus, 2001; Williams, Barnett, & Meck, 1990) and perform better than males on object location memory tasks (e.g., Saucier, Schulz, Keller, Cook, & Binsted, 2008), while males typically perform better than females in other tests of spatial ability (e.g., Jonasson, 2005; Seymoure, Dou, & Juraska, 1996). Therefore, from a proximate perspective, a greater reliance on landmarks or features might result in female rats spending more time exploring than males.

In this study, we compared the exploratory behavior of male and female rats in two novel environments during the adolescent period. We used the open field (*OF*) apparatus and the elevated plus-maze (*EPM*) apparatus as our novel environments. These tasks are commonly used to measure locomotor activity and anxiety-like responses in rodents (Prut & Belzung, 2003; Walf & Frye, 2007). The OF consists of a novel, enclosed space (Hall, 1934, 1936), while the EPM consists of two open and two enclosed arms arranged in a plus-shape and raised above the ground (Handley & Mithani, 1984; Montgomery, 1955). Both pieces of apparatus allow researchers to measure total locomotor activity and also provide information about the time spent in relatively aversive areas. The center of the OF and the open arms of the EPM are assumed to be aversive to rodents, on the basis that locomoting in open spaces potentially increases predation risk in the rodent's natural habitat (Pellow, Chopin, File, & Briley, 1985; Prut & Belzung, 2003). While some studies have reported that locomotor activity in the OF correlates highly with locomotor activity in the EPM (e.g., Lalonde & Strazielle, 2008), other evidence suggests that these two tests differentially elicit anxiety-like responses (Carola, D'Olimpio, Brunamonti, Mangia, & Renzi, 2002; Ho, Eichendorff, & Schwarting, 2002). Here, patterns of exploratory behavior in both tests will be compared.

Few studies have examined the ontogeny of exploratory behavior in adolescent rodents. An early study reported that the total amount of locomotion in the OF increases across the adolescent period in rats (Candland & Campbell, 1962). However, these researchers failed to report the amount of time spent by adolescent rats in the relatively aversive center of the OF, limiting the amount of information gained about exploration. In contrast to this study, two others have reported that locomotor activity in the OF declines across adolescence in rats (Masur, Schutz, & Boerngen, 1980; Philpot & Wecker, 2008). Studies of sex differences in OF activity during adolescence have been similarly inconsistent. Female adolescent rats have been reported to locomote more in an OF than same-aged males (Beatty & Fessler, 1976; Blizard, Lippman, & Chen, 1975; Fraňková & Barnes, 1968; Stewart, Skvarenina, & Pottier, 1975), while other studies have failed to find a sex difference in locomotion at this age (Masur et al., 1980; Selinger, 1977; Slob, Huizer, & van der Werff ten Boesch, 1986; Stevens & Goldstein, 1981). None of these studies reported time spent in the center of the OF by male and female adolescent rats or measured EPM performance in the same subjects. In the EPM, mid-adolescent female rats have been reported to spend more time on the open arms, or make a greater proportion of open arm entries, than males of the same age (Elliott, Faraday, Phillips, & Grunberg, 2004; Imhof, Coelho, Schmitt, Morato, & Carobrez, 1993; Leussis & Andersen, 2008). In contrast, another study suggested that this sex difference does not emerge until early adulthood (Estanislau & Morato, 2006). Detailed information on OF and EPM performance across the entire adolescent period in male and female rats is therefore currently lacking.

Here, we investigated the exploratory behavior of male and female rats across adolescence in both the OF and EPM tasks, including analyses of time spent in the more aversive areas of each apparatus. We predicted that exploratory behavior would increase across adolescence and set out to evaluate the predictions of alternative hypotheses regarding sex differences in exploration. By measuring performance in both the OF and EPM, we were able to test whether exploratory behavior differs between these two environments. Given that body weight varies across adolescence, and that behavioral changes might result from developmental changes in physical strength or motor coordination, we included body weight as a covariate in the analyses. We will discuss the possibility that developmental changes in neural function underlie age and sex differences in exploratory behavior across adolescence.

## METHODS

### Subjects and Housing

The subjects were 25 male and 21 female Lister-hooded rats (*Rattus norvegicus*), selected from four litters bred in-house (original stock acquired from Harlan, Blackthorn, UK). The subjects were housed in a holding room with reverse-lighting (lights on from 23:00-11:00; temperature: 20 ± 1°C; relative humidity: 55 ± 5%) in plastic and wire mesh homecages (52 cm × 40 cm × 26 cm). Water and soy-free pelleted food were available ad libitum. The offspring were removed from the natal cage on postnatal day 21 and housed with same-sex litter mates.

The offspring were assigned to one of the three adolescent age categories for testing, with 16 individuals (9 males, 7 females) in the *early adolescent* group, 16 individuals in the *mid-adolescent* groups (9 males, 7 females), and 14 individuals (7 males, 7 females) in the *late adolescent* group. The subjects in each group were balanced as closely as possible across the four litters. Each subject was only tested once in each novel environment, as repeated testing in the OF and EPM has been shown to affect performance (e.g., Bertoglio & Carobrez, 2000; Izídio, Spricigo, & Ramos, 2005). Given that body weight is predicted to differ across age and sex groups, all subjects were weighed at weekly intervals, so that we could include body weight as a covariate in the analyses of behavioral data.

All guidelines and requirements set out in the Principles of Laboratory Animal Care (National Institutes of Health, U.S.A., Publication No. 86-23, revised 1985) and the U.K. Animals (Scientific Procedures) Act 1986 were followed.

### Apparatus and Experimental Design

The animals completed the tests in the following order: the OF and the EPM. All animals were tested in the same order on the tests, so that any possible order effects were uniformly distributed across all groups. To further reduce the possibility that testing in one apparatus would influence the performance on the other apparatus, a period of one week separated the testing days. OF testing was carried out at 24–26 days (early adolescents), 38–40 days (mid-adolescents), or 52–54 days (late adolescents), and EPM testing was carried out at 30–32 days (early adolescents), 44–46 days (mid-adolescents), and 58–59 days (late adolescents). Tests were conducted between 10:30 and 14:30 hr in a testing room under dim, white light (approximately 25 lux). Details of apparatus design are provided below:

- The OF consisted of an area of hard vinyl floor (120 cm 120 cm) enclosed on four sides by a gray, wooden wall (50 cm high). The floor area was marked into nine areas (eight outer and one central area) by drawing four lines with red pen, each 30 cm from one of the walls. At the beginning of the test, the subject was placed into the front left corner of the OF and observed for 10 min. After each test, the apparatus was cleaned with 70% alcohol to remove any odor cues.
- The EPM consisted of four gray, wooden arms (51cm long × 11 cm wide) raised 56 cm from the ground on a metal frame. Two of the arms had walls (closed arms; 40 cm high) and the remaining two arms lacked walls (open arms). At the start, the subject was placed into the central area, facing a closed arm, and each test lasted 5 min. After each test, the apparatus was cleaned with 70% alcohol to remove any odor cues.

### Behavioral Measurements

During each OF or EPM test, behavior data were recorded directly onto a laptop computer running in-house software. The inter-rater reliability between the two observers (D.L. and G.B.) was confirmed to be greater than 90%.

In the OF, the animal was recorded as entering a new area when all four of the animal's paws crossed the boundary into a different marked-out area. In the EPM, entering a new area was recorded when all four paws crossed onto a new arm or into the central area. From these measures, the following scores were calculated: (i) *total locomotion in the OF or EPM* (total number of line crossings in the OF; total number of entries into closed arms, open arms and central area in the EPM), (ii) *percentage of entries into the center of the OF* (total entries into the area ÷ total locomotion × 100) and (iii) *percentage of time spent on the open arms of the EPM* (total time spent in the area ÷ total duration of test × 100).

### Statistical Analyses

For the OF and EPM data, normal and normalized (log-transformed) data were analyzed using separate multivariate ANOVAs for each test, with age, sex, and litter as between-subject factors. As no significant main effects of litter, or interactions between litter and age or litter and sex, were found, these results are not reported. Post hoc Scheffe's tests were performed where appropriate. The weight data were subject to a repeated-measures ANOVA with sex as a between-subject factor and age as a within-subject factor. The behavioral data were also analyzed using an analysis of covariance (ANCOVA) with body weight as a covariate. Pearson's correlation coefficient tests were used to examine the relationship between measures on the two behavioral tests. All data are reported as means ± standard errors (*SEMs*).

## RESULTS

### Body Weight

Main effects of age (*F*_5,220_ = 80.68, *p* < .001) and sex (*F*_1,44_ = 25.00, *p* < .001) were found for body weight, with weight increasing with age and males weighing more than females. The interaction between age and sex was significant (*F*_5,220_ = 3.07, *p* = .011), due to males gaining weight more quickly than females across the adolescent period.

### Open Field

#### Total Locomotion

**FIGURE 1:**
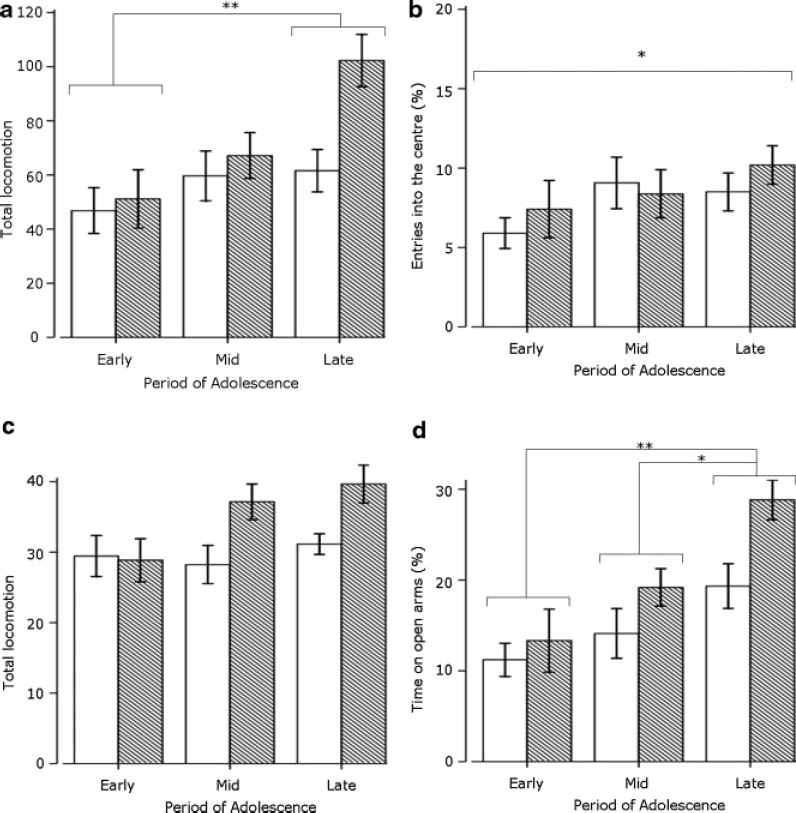
(a) Open field total locomotion, (b) percentage of center entries in the open field, (c) elevated plus-maze total locomotion, (d) percentage duration on open arms of the elevated plus-maze (means and SEMs). *p < .05, **p < .01 indicate a significant difference in post hoc test, except for (b) where *p < .05 indicates a main effect of age. White bars represent males, hatched bars represent females.

Total locomotion in the OF differed significantly between age groups (*F*_2,22_ = 6.67, *p* = .005) and between sexes (*F*_1,22_ = 7.62, *p* = .011), with locomotion increasing with age and females locomoting more than males. Post hoc tests revealed that late adolescents locomoted more than early adolescents (Fig. 1a). The main effects of age on total locomotion persisted when body weight was included as a covariate (*F*_2,21_ = 6.96, *p* = .005), with post hoc comparisons again revealing that late adolescents locomoted more than early adolescents (*p* = .003). Although the interaction between age and sex was not significant (*F*_3,22_ = 1.10, *p* = .351), the age and sex effects appear to be strongly influenced by the high levels of locomotion exhibited by late adolescent females.

#### Percentage of Entries into the Center

The percentage of entries into the center differed with age group (*F*_2,22_ = 3.86, *p* = .036; Fig. 1b) but not with sex (*F*_1,22_ = .92, *p* = 349). Post hoc tests using pair-wise comparisons did not locate significant differences between the age groups. No significant interaction was found between age and sex (*F*_3,22_ = .87, *p* = .345). The main effect of age persisted after covarying body weight (*F*_2,21_ = 3.81, *p* = .039), with pair-wise comparisons showing that late adolescents made a higher percentage of center entries than early adolescents (*p* = .029).

### Elevated Plus Maze

#### Total Locomotion

Total locomotion in the EPM tended to increase with age (*F*_2,22_ = 2.63, *p* = .085) and differed significantly between the sexes (*F*_1,22_ = 6.48, *p* = .015; Fig. 1c), with females locomoting more than males. After including weight as a covariate in the analysis, the effect of age on total locomotion remained as a trend (*F*_2,42_ = 2.78, *p* = .074). Although no significant interaction was found between age and sex (*F*_3,22_ = 2.06, *p* = .141), the effect of age appears to be strongly influenced by an increase in locomotion with age in females rather than males.

#### Percentage of Time on Open Arms

The percentage of time spent on the open arms differed significantly between the age groups (*F*2,22 = 10.47, *p* < .001) and between sexes (*F*_1,22_ = 7.15, *p* = .011; Fig. 1d), with time spent on the open arms increasing across adolescence and being higher for females than males. Post hoc analyses revealed that late adolescents had higher scores on this measure than both early and mid-adolescents. Using weight as a covariate, the main effect of age persisted (*F*_2,41_ = 6.02, *p* = .005), with post hoc comparisons revealing that all three age groups differed from each other, with time on the open arms increasing with age (early vs. mid-adolescents: *p* = .049, early vs. late adolescents: *p* = .005, mid- vs. late adolescents: *p* = .007). No significant interaction was found between sex and age (*F*_3,22_ = 1 .02, *p* = .370).

### Correlations between OF and EPM Performance

A significant positive correlation was found between total locomotion in the OF and total locomotion in the EPM (*r* = .324, *p* = .030). The percentage of entries into the center of the OF tended to correlate positively with the percentage of time spent on the open arms of the EPM (*r* = .268, *p* = .076).

## DISCUSSION

The aim of this study was to describe the ontogeny of exploratory behavior across adolescence in male and female rats. The results indicate that (i) general locomotor activity increases across adolescence and (ii) amount of exploration directed towards aversive areas of the novel environments increases across adolescence. We conclude that, in support of our first prediction, exploratory behavior in novel, potentially risky environments increases across adolescence in rats. An increase in active attempts to gain information about the environment during adolescence might function to promote dispersal and the transition to independence in wild rats. With regards to sex differences, the results showed that female adolescents locomote more than males in both the OF and EPM, and that females spend more time on the aversive open arms of the EPM than males. Here, we compare our results with previous findings and discuss the possible alternative explanations for age and sex differences in exploratory behavior across adolescence in rats.

Our finding that total locomotor activity in the OF increased across the adolescent age groups confirms an early study of adolescent rats (Candland & Campbell, 1962), but contradicts two later studies, which reported that locomotor activity declines across adolescence in rats (Masur et al., 1980; Philpot & Wecker, 2008). One of these studies (Masur et al., 1980) tested the same subjects successively at different ages, thereby potentially confounding any age effects with habituation effects, while, in the other (Philpot & Wecker, 2008), subjects were handled twice daily for 3 days prior to testing—experimenter handling has been shown to influence locomotor behavior in the OF (e.g., Denenberg, 1969; Vallée et al., 1997; Williams & Russell, 1972). In line with previous studies (e.g., Beatty & Fessler, 1976; Blizard et al., 1975), we found that female adolescent rats locomote more in an OF than same-aged males. In fact, the age differences in locomotor activity appear to be strongly influenced by the high levels of locomotor activity in late adolescent females, with locomotion remaining relatively constant across adolescence for males.

Our results also showed that the percentage of entries into the center of the OF and percentage of time spent on the open arms of the EPM significantly increased across adolescence. While little comparable data has been reported for rats, these results support a previous study of mice, which reported that time spent on the open arms of an EPM increased from early adolescence to early adulthood (Hefner & Holmes, 2007). In our study, females spent more time on the open arms of the EPM than males. The lack of a significant interaction between age and sex prevented us from examining the age at which this sex difference emerges. Previous studies of rats have either reported that the sex difference in open arm activity is present at mid-adolescence (Elliott et al., 2004; Imhof et al., 1993; Leussis & Andersen, 2008) or reported that the sex difference does not emerge until early adulthood (Estanislau & Morato, 2006). Either way, the sex differences in EPM performance that are reported in adult rats (e.g., Aguilar et al., 2003; Johnston & File, 1991) apparently emerge gradually during the adolescent period.

Comparing the performance of subjects across the two tests indicates that locomotor activity in the OF correlates positively with locomotor activity in the EPM, and time spent in the aversive areas of each apparatus tended to correlate, such that individuals with high scores on one test generally have high scores on the other. This supports previous studies that have found a correlation between measures of locomotion in these two tests (e.g., Lalonde & Strazielle, 2008) and suggests that both pieces of apparatus elicit similar responses in individual rats.

The increase in exploratory behavior across adolescence could potentially be explained in terms of the animals becoming more physically capable of locomoting around their environments as they mature. During adolescence, rats gain physical strength and motor coordination (Brown, 2005). However, when body weight was included as a covariate in the analyses, the effect of age on total locomotion in the OF, proportion of entries into the center of the OF, and time spent on the open arms of the EPM remained significant, suggesting that physical development (at least as estimated by body weight) does not completely explain the observed changes in behavior across adolescence.

An alternative potential explanation is that changes in exploratory behavior across adolescence are underlain by changes in the functioning of the central nervous system. Adolescence is also known to be a period of development during which the brain is undergoing widespread changes (Blakemore & Choudhury, 2006; Crews, He, & Hodge, 2007; Spear, 2000b), including brain systems involved in motivation and emotional responses that might play a role in exploratory behavior. Growing evidence suggests that changing levels of steroid hormones play a role in the changes brain function and behavior during adolescence (McCormick & Mathews, 2007; Sisk & Zehr, 2005). Thus, the increased amount of locomotion and proportion of time spent in aversive areas of novel environments across adolescence in rats might be related to changes in the neuroendocrine systems involved in fearfulness, anxiety-like behavior, attraction to novelty, and risk-taking behavior. Similarly, male and female adolescent rodents might differ in anxiety-like responses, motivation to explore novel environments, willingness to take risks, or ability to remember aspects of their environment. Understanding the mechanisms underlying changes in exploratory behavior across adolescence and between the sexes in rats could increase our understanding of the links between hormones, behavioral development, and brain function in human adolescence.

## NOTES

This research was funded by the Wellcome Trust (Research Career Development Fellowship awarded to G.B.), the Association for the Study of Animal Behaviour (Undergraduate Project Scholarship awarded to D.L.), and the School of Psychology, University of St Andrews. We thank the technical staff in the School of Psychology for support, and Kevin Laland and two anonymous reviewers for comments on the manuscript.
